# Assessing the quality of online resources for vascular neurosurgery and skull base surgery: A bilingual evaluation using DISCERN and JAMA benchmarks

**DOI:** 10.1016/j.bas.2026.107718

**Published:** 2026-11-02

**Authors:** Janek Garber, Julia Bettina Kräutler, Daniel Monden, Nadine Lilla, Felix Behling, Florian Gessler, Ansgar Berlis, Christian Senft, Dorothee Mielke, Paulina Günzerodt, Peter Baumgarten

**Affiliations:** a Department of Neurosurgery, University Hospital Jena, Friedrich Schiller University, Jena, Germany; b Department of Neurosurgery, Faculty of Medicine, University of Augsburg, Augsburg, Germany; c Department of Neurosurgery, University Medicine Rostock, Rostock, Germany; d Diagnostic and Interventional Neuroradiology, Faculty of Medicine, University of Augsburg, Augsburg, Germany

**Keywords:** Online health information, Vascular neurosurgery, Skull base surgery, DISCERN, JAMA benchmarks, Patient education

## Abstract

**Introduction:**

Patients increasingly seek information about complex neurosurgical conditions online. The quality of such resources has not been systematically studied for vascular neurosurgery and skull base surgery.

**Research question:**

How do German- and English-language online resources for eight vascular and skull base neurosurgical conditions compare in reliability and transparency, and is search rank associated with quality?

**Material and methods:**

A simulated Google search was performed for nine search terms covering eight conditions in German and English. The first 20 results per search were rated by two independent raters using the modified DISCERN Plus score (16–80) and JAMA benchmarks (0–4). Non-parametric tests, effect sizes and intraclass correlation coefficients were calculated.

**Results:**

In total, 355 websites were included (177 English, 178 German). Mean DISCERN score was 41.1 ± 9.7 and mean JAMA score 2.61 ± 0.94 (fair quality). English-language sites scored higher than German sites on both instruments, with small effect sizes (r = 0.12–0.13). Host type was strongly associated with JAMA scores (ε^2^ = 0.25); educational and governmental sites scored highest. Among English-language results, top-ranked websites were of higher quality (rho = −0.315; positions 1–5: median DISCERN 49.8 vs. 40.0–42.0 thereafter), whereas rank was unrelated to quality in German-language results. Interrater agreement was good to excellent (ICC 0.79–0.82).

**Discussion and conclusion:**

Online resources for these conditions are generally of fair quality. Top-ranked English-language results tended to be of higher quality, whereas search rank was not informative for German-language results. Guiding patients, particularly German-speaking patients, towards reliable resources remains essential.

## Highlights

• Online resources for vascular and skull base neurosurgery are of fair quality.
• English websites scored slightly higher than German ones on DISCERN and JAMA.
• Educational and governmental sites scored highest; host type drove JAMA scores.
• Top-ranked English results were of higher quality; rank was uninformative in German.
• Findings support guiding patients to vetted online neurosurgical resources.

## 1 Introduction

The internet has profoundly changed how patients and their relatives obtain information about medical conditions. A growing proportion of individuals seek health information online prior to or following medical consultations, and this behavior is particularly pronounced in the context of serious or complex diagnoses (Moccia et al., 2018). The quality of web-based resources, however, is neither regulated nor standardized, and discrepancies between scientifically accurate information and what is actually available online have been documented across many medical specialties (Liang et al., 2011).

Vascular neurosurgery and skull base surgery encompass conditions that are both life-threatening and technically challenging, for example: intracranial aneurysms, subarachnoid hemorrhage, cerebral arteriovenous malformations (AVMs), dural arteriovenous fistulas (dAVFs), cerebral cavernomas, Moyamoya disease, vestibular schwannomas (still widely referred to as acoustic neuromas), and skull base chordomas. These conditions are often associated with substantial uncertainty about prognosis and treatment, making access to accurate patient information particularly important. Patients facing such diagnoses frequently have limited time to talk to their treating physicians and may rely heavily on what they find online when processing their diagnosis or weighing treatment options (Boogaarts et al., 2014; Broomfield and O'Donoghue, 2016).

Several tools have been developed to assess the quality of health information, of which the DISCERN instrument (Charnock et al., 1999) and the JAMA benchmarks (Silberg et al., 1997) are the most widely used in neurosurgical research. The DISCERN score evaluates the reliability of health information and the quality of treatment-related content across 16 items rated on a five-point Likert scale (maximum 80 points) (Charnock, 1998), while the JAMA benchmarks assess four key transparency criteria - authorship, attribution of sources, currency, and disclosure of ownership (maximum 4 points) (Silberg et al., 1997).

Published studies using these instruments have focused primarily on selected vascular conditions. Several groups have evaluated online resources for intracranial aneurysms (Patel et al., 2022) and cerebral AVMs (Mehra et al., 2023) and found quality to be consistently in the low-to-moderate range. Readability studies have further highlighted that a substantial portion of cerebrovascular online content is written above the recommended reading level for health materials (Gajjar et al., 2024a; Edwards et al., 2022). For skull base conditions, systematic quality assessments are largely lacking. Patient perception of cavernous malformation online information has been addressed in the context of social media (Gajjar et al., 2024b) and patient-reported outcome data exist for vestibular schwannoma (Broomfield and O'Donoghue, 2016) and skull base chordoma (Myneni et al., 2025), but formal quality scoring of general web resources for these entities has not been reported.

An additional dimension relevant to the German healthcare context is the language of available resources. Previous work has shown that German-language health web content is generally less readable and of lower quality than its English-language counterpart (Möller et al., 2023; Meyer et al., 2014; Zowalla et al., 2023), yet most prior quality assessments have focused exclusively on English-language sources (Patel et al., 2022; Mehra et al., 2023).

The objective of this study was to systematically evaluate the quality of German- and English-language online patient information for eight vascular and skull base neurosurgical conditions, searched under nine commonly used terms, using standardized instruments, and to identify factors that are associated with higher information quality, including the position of a website in the search results.

## 2 Materials and methods

### 2.1 Search strategy and website selection

A simulated Google search was performed independently for each of nine search terms, covering eight distinct conditions, in both German and English (Table 1). Searches were performed between March and May 2026. The German-language search terms were: Akustikusneurinom, Vestibularisschwannom, Chordom Schädelbasis, Hirnaneurysma, Subarachnoidalblutung, arteriovenöse Malformation Gehirn, durale arteriovenöse Fistel, Kavernom Gehirn, and Moya-Moya-Krankheit. The English-language equivalents were: acoustic neuroma, vestibular schwannoma, skull base chordoma, intracranial aneurysm, subarachnoid hemorrhage, cerebral arteriovenous malformation, dural arteriovenous fistula, cerebral cavernoma, and Moyamoya disease. Searches were conducted in private (incognito) browsing mode without active login to minimize personalization effects. The browser cache was cleared before the search and a VPN was used to set the location to Berlin, Germany for German-language searches and to New York City, USA for English-language searches. The first 20 results for each language-search term combination were selected, yielding 40 websites per search term and 360 websites in total. The position of each website in the result list was recorded as its Google search rank (1 = first result, 20 = twentieth result).

**Table 1:** Sample characteristics by host type and language.

Host type	English, n (%)	German, n (%)	All, n (%)
University/medical center	95 (53.7)	86 (48.3)	181 (51.0)
Educational	34 (19.2)	45 (25.3)	79 (22.3)
Interest group/NGO	23 (12.9)	20 (11.2)	43 (12.1)
Medical news	3 (1.7)	23 (12.9)	26 (7.3)
Governmental	21 (11.9)	3 (1.7)	24 (6.8)
Other	1 (0.6)	1 (0.6)	2 (0.6)
All	177 (100.0)	178 (100.0)	355 (100.0)

Websites were excluded if they were inaccessible (broken link, paywall, login requirement) or if they provided no evaluable health information (e.g., image databases, pure news aggregators without substantive content). Sites where both the DISCERN and JAMA scores were zero for both raters were removed from the analysis. Following exclusion, 355 websites were available for analysis.

### 2.2 Quality assessment instruments

Each website was independently assessed by two raters using the modified DISCERN Plus score and the JAMA benchmarks. The modified DISCERN Plus instrument consists of 16 items each rated on a five-point scale (1 - 5), yielding a maximum possible score of 80 (Charnock et al., 1999; Rees et al., 2002). We used the modified DISCERN Plus score analogous to Baumgartner et al. (2024) In accordance with the classification proposed by Nghiem et al., scores were categorized as: very poor (<18), poor (18 - 34), fair (35 - 50), good (51 - 67), and excellent (≥68) (Khazaal et al., 2009; Nghiem et al., 2016). The JAMA benchmarks assess four binary criteria: authorship (is the author identified?), attribution (are sources referenced?), currency (is the content dated?), and disclosure (is ownership and potential conflict of interest disclosed?). Each fulfilled criterion adds one point, giving a maximum of 4 (Silberg et al., 1997).

Both raters assessed all websites independently and were based at different institutions: J.G. was a final-year medical student and doctoral candidate at the Department of Neurosurgery, University Hospital Jena, and P.G. was a neurosurgical resident in the third year of training at the Department of Neurosurgery, University Hospital Augsburg. Both raters used the standardized assessment chart provided in Supplementary File S1. Of note, DISCERN was explicitly developed for use by health consumers and information providers without specialist knowledge, and the JAMA benchmarks are formal transparency criteria; neither instrument assesses the clinical accuracy of the content against guideline recommendations (Charnock et al., 1999; Silberg et al., 1997). For each website the mean of the two raters' scores was used for all analyses.

The search terms acoustic neuroma and vestibular schwannoma denote the same entity. Although vestibular schwannoma is the correct terminology, the term acoustic neuroma is still widely used by patients and was therefore searched separately to reflect real-world search behavior. Because the two searches generated partially different result lists (6 of 19 German-language and 8 of 20 English-language websites were retrieved by both terms), both terms are presented separately in the descriptive results; a sensitivity analysis with both terms pooled into a single condition is reported in Section 3.3.

### 2.3 Website classification

Each included website was classified into one of six host categories based on the primary affiliation of the website owner: university hospital or clinic (including academic medical centers and teaching hospitals), educational website (health information portals and patient education platforms affiliated with medical societies or professional bodies), interest group or NGO (patient advocacy organizations, self-help groups, and disease-specific foundations), medical news (online health journalism and medical news platforms), governmental website (national or regional health authorities and ministries), and other (websites not fitting into the above categories).

### 2.4 Statistical analysis

The Shapiro-Wilk test was used to assess the distribution of scores. As neither DISCERN nor JAMA scores were normally distributed (DISCERN: W = 0.972, p < 0.001; JAMA: W = 0.928, p < 0.001), all group comparisons were performed using non-parametric tests. The Mann-Whitney *U* test was applied to compare English- and German-language websites. Differences across conditions and across website host categories were assessed using the Kruskal-Wallis H test with Bonferroni correction for post-hoc pairwise comparisons.

The association between Google search rank and website quality was evaluated using the Spearman rank correlation coefficient. Search rank was entered as the position in the result list (1 = first result); a negative coefficient therefore indicates that quality scores decrease with increasing rank position, i.e., that websites placed higher in the result list have higher scores. In addition, websites at positions 1 - 10 were compared with those at positions 11 - 20 using the Mann-Whitney *U* test. The correlation between DISCERN and JAMA scores was also assessed with Spearman's rho. Interrater reliability was quantified using the intraclass correlation coefficient (ICC) based on a two-way random-effects model with absolute agreement [ICC(2,1)], following the approach described by Shrout and Fleiss.

Effect sizes are reported alongside p-values: the correlation coefficient r (r = |Z|/√N) for Mann-Whitney U tests, epsilon-squared (ε^2^ = H/(n - 1)) for Kruskal-Wallis tests, and Spearman's rho for correlations. Following common conventions, r values of approximately 0.1, 0.3 and 0.5 and ε^2^ values of approximately 0.01, 0.06 and 0.14 were interpreted as small, medium and large effects, respectively (Fritz et al., 2012).

All analyses were performed using JMP® Edition 19, JMP Statistical Discovery LLC, Cary, NC, USA. Statistical significance was set at α = 0.05 (two-tailed). Where multiple comparisons were performed, Bonferroni-corrected thresholds are reported.

## 3 Results

### 3.1 Overall score distribution

After exclusion of five websites, 355 websites were included in the final analysis (177 English, 178 German). The mean DISCERN score across all websites was 41.1 ± 9.7 (median 42.0, IQR 35.5 - 48.5, range 16 - 63) and the mean JAMA score was 2.61 ± 0.94 (median 2.5, IQR 2.0 - 3.5, range 1 - 4). Using the five-category Nghiem classification the majority of websites fell into the “fair” quality range (63.4%, n = 225), followed by “poor” (20.3%, n = 72) and “good” (15.5%, n = 55). No website achieved an “excellent” rating, and only 3 websites (0.8%) were classified as “very poor” (Fig. 2, Table 2).

**Table 2:** DISCERN and JAMA scores by language, condition, and host type.

Stratum	n	DISCERN, median (IQR)	DISCERN, mean ± SD	JAMA, median (IQR)
**Language**				
English	177	43.0 (36.5–49.0)	42.6 ± 9.1	2.5 (2.0–3.5)
German	178	41.0 (33.6–47.5)	39.7 ± 10.0	2.5 (2.0–3.5)
**Condition (search term)**				
Acoustic neuroma	39	43.5 (37.8–50.0)	43.8 ± 9.0	2.5 (2.0–3.5)
Vestibular schwannoma	40	43.2 (36.9–48.4)	42.1 ± 9.9	2.5 (2.0–3.5)
Skull base chordoma	39	42.0 (37.2–46.8)	41.1 ± 9.7	3.0 (2.0–3.5)
Intracranial aneurysm	39	43.5 (37.5–48.5)	42.4 ± 10.2	2.5 (2.0–3.5)
Subarachnoid hemorrhage	40	46.8 (37.8–50.8)	44.0 ± 9.2	3.0 (2.5–3.5)
Cerebral AVM	39	43.5 (35.8–49.0)	41.9 ± 7.9	2.5 (2.0–3.5)
Dural AV fistula	39	37.5 (31.8–47.0)	38.6 ± 10.4	2.5 (2.0–3.5)
Cerebral cavernoma	40	39.0 (32.1–47.1)	38.3 ± 10.5	2.0 (1.5–3.0)
Moyamoya disease	40	39.2 (33.9–44.2)	38.3 ± 8.6	3.0 (2.5–3.5)
**Host type**				
University/medical center	181	41.0 (35.5–48.0)	41.5 ± 8.6	2.0 (2.0–3.0)
Educational	79	46.0 (38.0–49.2)	42.4 ± 9.7	3.5 (3.0–4.0)
Interest group/NGO	43	40.5 (35.0–43.5)	38.1 ± 9.8	2.5 (2.0–3.0)
Medical news	26	41.0 (30.0–47.8)	37.7 ± 12.6	3.0 (2.0–4.0)
Governmental	24	44.5 (41.4–50.0)	43.7 ± 10.0	3.0 (3.0–4.0)
Other	2	36.2 (27.4–45.1)	36.2 ± 25.1	3.2 (2.9–3.6)

DISCERN total score ranges from 16 to 80; the modified score reported here uses the mean of two raters. JAMA score ranges from 0 to 4. IQR, interquartile range; SD, standard deviation; AVM, arteriovenous malformation; AV, arteriovenous.

### 3.2 Comparison by language

English-language websites scored significantly higher than German-language websites on both instruments. The median DISCERN score was 43.0 (IQR 36.5 - 49.0) for English and 41.0 (IQR 33.6 - 47.5) for German sites (Mann-Whitney U = 18205.0, p = 0.011, r = 0.13). The median JAMA score was 2.5 in both language groups, but the distribution differed significantly (Mann-Whitney U = 17829.0, p = 0.030, r = 0.12). Both effects were small in magnitude.

When quality categories were compared by language, a higher proportion of English-language sites achieved a “good” rating (20.3%, n = 36) compared to German sites (10.7%, n = 19), whereas German sites showed a higher proportion of “poor” ratings (25.8%, n = 46 vs. 14.7%, n = 26 for English). The distribution is shown in Fig. 2.

### 3.3 Differences across conditions

Statistically significant differences in both DISCERN scores (Kruskal-Wallis H = 18.86, p = 0.016, ε^2^ = 0.053) and JAMA scores (H = 19.42, p = 0.013, ε^2^ = 0.055) were found across the nine search terms. Subarachnoid hemorrhage showed the highest median DISCERN score (46.8, IQR 37.8 - 50.8), while dural AV fistula, cerebral cavernoma, and Moyamoya disease scored lowest (medians 37.5, 39.0, and 39.2, respectively). Skull base chordoma, subarachnoid hemorrhage, and Moyamoya disease had the highest median JAMA scores (3.0 each).

After Bonferroni correction (adjusted α = 0.0014 for 36 pairwise comparisons), none of the pairwise differences reached statistical significance. Together with the small effect sizes, this suggests that the overall Kruskal-Wallis result reflects a distributed pattern of variation across conditions rather than a single dominant outlier. Results by condition are displayed in Fig. 1 and Table 2.

**Fig. 1:**
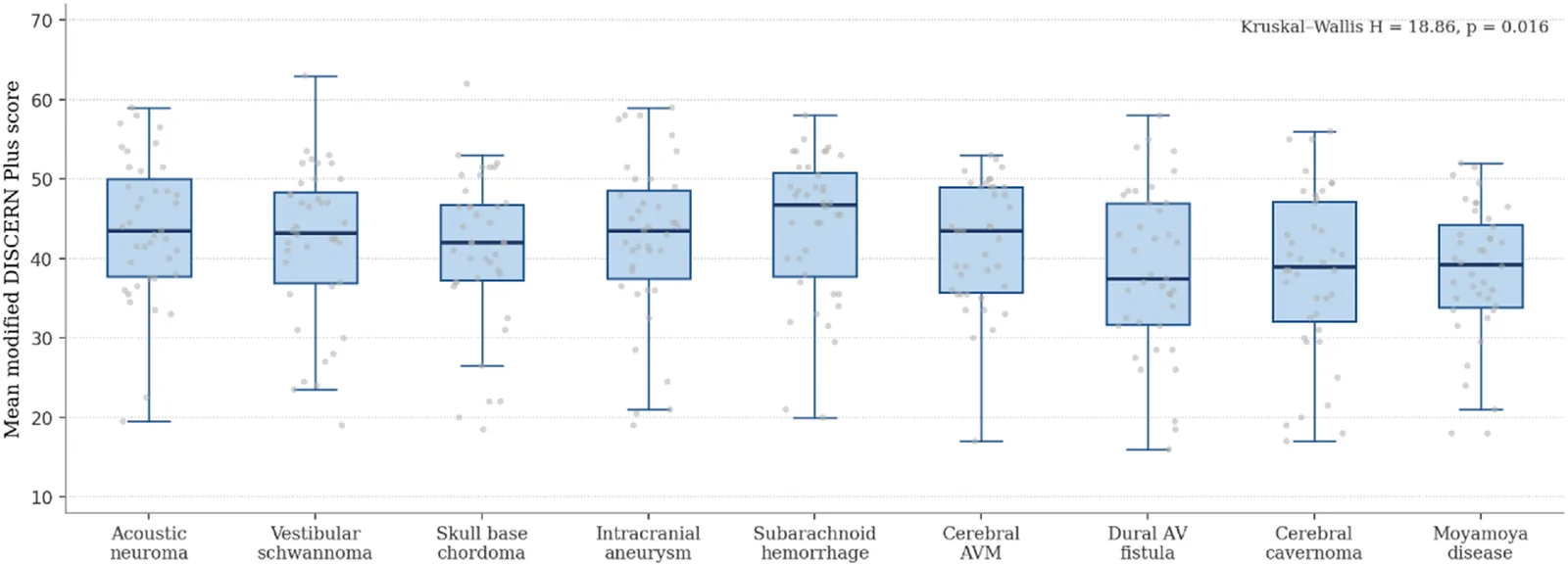
Distribution of mean modified DISCERN Plus scores by neurosurgical condition. Boxes show the median and interquartile range; whiskers show the 1.5 × IQR range; dots indicate individual websites. Significant differences across conditions were found (Kruskal-Wallis H = 18.86, p = 0.016). AVM, arteriovenous malformation; AV, arteriovenous.

**Fig. 2:**
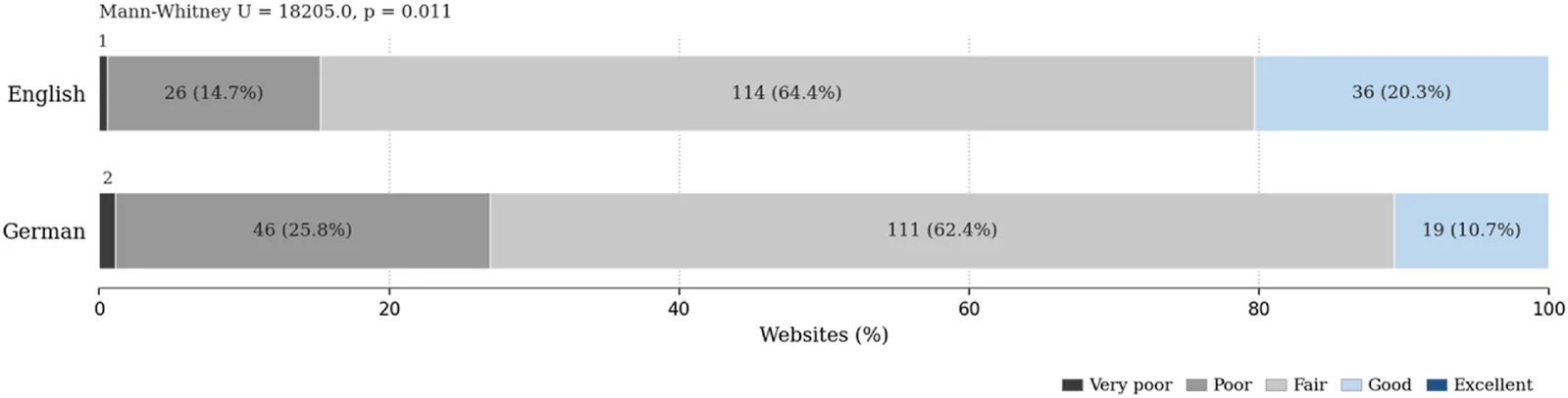
Distribution of DISCERN quality categories by language. Categories follow the five-tier classification adapted from Nghiem et al. 'Very poor' ratings were more common in German-language websites, while 'good' ratings were more common in English-language websites (Mann-Whitney U = 18205.0, p = 0.011). Numbers within bars indicate absolute counts. No website achieved an 'excellent' rating.

The two search terms for the same entity, acoustic neuroma and vestibular schwannoma, yielded nearly identical scores (median DISCERN 43.5 vs. 43.2, Mann-Whitney U p = 0.610; median JAMA 2.5 vs. 2.5, p = 0.538). In a sensitivity analysis in which both terms were pooled into a single condition, the differences across the resulting eight conditions remained significant (DISCERN: H = 18.53, p = 0.010, ε^2^ = 0.052; JAMA: H = 19.09, p = 0.008, ε^2^ = 0.054).

### 3.4 Differences by website host category

A significant difference was found for JAMA scores (H = 87.68, p < 0.001, ε^2^ = 0.248, corresponding to a large effect), while the difference in DISCERN scores did not reach significance (H = 10.99, p = 0.052, ε^2^ = 0.031) across the six host categories. University and clinic websites represented the largest group (51.0%, n = 181) and had a median DISCERN score of 41.0 (IQR 35.5 - 48.0). Educational websites (22.3%, n = 79) achieved the highest median DISCERN score (46.0, IQR 38.0 - 49.2). Educational sites had the highest median JAMA score (3.5), followed by governmental and medical news sites (3.0 each), while university and clinic websites scored notably lower on JAMA (median 2.0).

Post-hoc analysis with Bonferroni correction revealed significant JAMA score differences between university/clinic sites and educational sites (p < 0.001, r = 0.51), university/clinic sites and medical news sites (p = 0.001, r = 0.23), university/clinic sites and governmental sites (p < 0.001, r = 0.27), educational sites and interest groups/NGOs (p < 0.001, r = 0.59), and interest groups/NGOs and governmental sites (p = 0.001, r = 0.40). No significant pairwise differences were detected for DISCERN scores after correction. Host category data are summarized in Table 3 and Fig. 3.

**Table 3:** Statistical comparisons.

Comparison	Metric	Test (statistic)	p-value	Effect size
German vs English	DISCERN	Mann-Whitney U = 18205.0	0.011	r = 0.13
German vs English	JAMA	Mann-Whitney U = 17829.0	0.030	r = 0.12
Across 9 search terms	DISCERN	Kruskal-Wallis H = 18.86	0.016	ε^2^ = 0.053
Across 9 search terms	JAMA	Kruskal-Wallis H = 19.42	0.013	ε^2^ = 0.055
Across host types	DISCERN	Kruskal-Wallis H = 10.99	0.052	ε^2^ = 0.031
Across host types	JAMA	Kruskal-Wallis H = 87.68	<0.001	ε^2^ = 0.248
Google rank (English)	DISCERN	Spearman ρ = −0.315	<0.001	ρ (medium)
Google rank (English)	JAMA	Spearman ρ = −0.178	0.018	ρ (small)
Google rank (German)	DISCERN	Spearman ρ = −0.081	0.284	ρ (small)
Google rank (German)	JAMA	Spearman ρ = −0.066	0.380	ρ (small)
Google rank (combined)	DISCERN	Spearman ρ = −0.191	<0.001	ρ (small)
Google rank (combined)	JAMA	Spearman ρ = −0.119	0.025	ρ (small)
Rank 1–10 vs 11–20 (English)	DISCERN	Mann-Whitney U = 4974.0	0.002	r = 0.23
Rank 1–10 vs 11–20 (German)	DISCERN	Mann-Whitney U = 3816.5	0.676	r = 0.03
Acoustic neuroma vs vestibular schwannoma	DISCERN	Mann-Whitney U = 832.5	0.610	r = 0.06
Across 8 conditions (pooled)	DISCERN	Kruskal-Wallis H = 18.53	0.010	ε^2^ = 0.052
Across 8 conditions (pooled)	JAMA	Kruskal-Wallis H = 19.09	0.008	ε^2^ = 0.054
DISCERN vs JAMA correlation	Score	Spearman ρ = 0.450	<0.001	ρ (medium)

Significance thresholds: for host-type comparisons, Bonferroni-corrected α = 0.0083 (six groups); for condition comparisons, α = 0.0014 (nine conditions). n.s., not significant; ρ, Spearman rank correlation coefficient. Google search rank was coded as the position in the result list (1 = first result); a negative ρ therefore indicates decreasing quality scores with increasing rank position, i.e., higher-placed websites had higher scores. Effect sizes: r = |Z|/√N for Mann-Whitney U tests (small ≈ 0.1, medium ≈ 0.3, large ≈ 0.5); ε2 = H/(n − 1) for Kruskal-Wallis tests (small ≈ 0.01, medium ≈ 0.06, large ≈ 0.14); Spearman ρ is itself an effect size. “Across 8 conditions (pooled)" refers to the sensitivity analysis in which acoustic neuroma and vestibular schwannoma were combined.

**Fig. 3:**
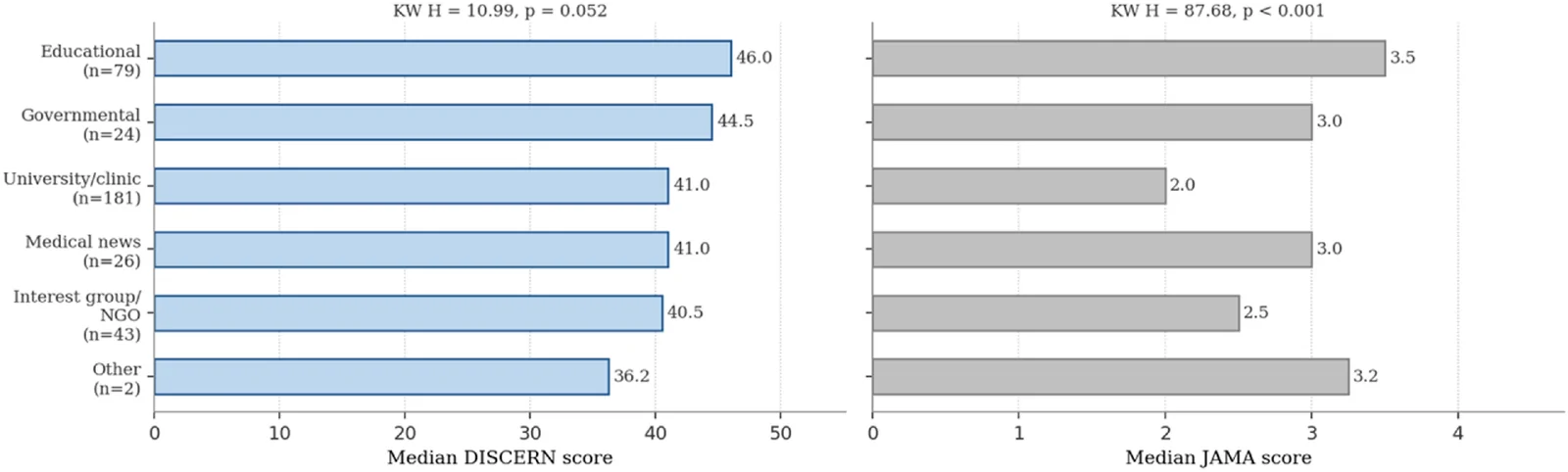
Median DISCERN and JAMA scores by website host category. Educational and governmental websites displayed the highest median scores on both instruments. The host-type effect was strongest for JAMA transparency (Kruskal-Wallis H = 87.68, p < 0.001). NGO, non-governmental organization.

### 3.5 Google search rank and information quality

Google search rank was coded as the position of a website in the result list (1 = first result). A significant negative correlation between Google search rank position and DISCERN score was found for English-language results (Spearman rho = −0.315, p < 0.001), indicating that websites placed higher in the search results (i.e., with lower rank numbers) tended to achieve higher DISCERN scores. A weaker but still significant association in the same direction was observed for JAMA (rho = −0.178, p = 0.018). In contrast, no significant correlation between rank position and quality was found for German-language results (DISCERN: rho = −0.081, p = 0.284; JAMA: rho = −0.066, p = 0.380).

For the combined cohort, the association remained statistically significant for both DISCERN (rho = −0.191, p < 0.001) and JAMA (rho = −0.119, p = 0.025), corresponding to a medium effect for DISCERN in English-language results and small effects otherwise.

Descriptively, English-language websites at positions 1 - 5 had a median DISCERN score of 49.8 (IQR 43.9 - 53.5), compared with 42.0 (IQR 35.5 - 47.0), 40.0 (IQR 33.8 - 47.8) and 41.0 (IQR 37.5 - 47.0) at positions 6 - 10, 11 - 15 and 16 - 20, respectively. English-language websites within the top 10 results scored higher than those at positions 11 - 20 (median 45.5 vs. 41.0; Mann-Whitney U = 4974.0, p = 0.002, r = 0.23). For German-language websites, the corresponding medians were 44.0, 35.5, 42.0 and 41.8, with no difference between the top 10 and positions 11 - 20 (median 40.5 vs. 42.0; U = 3816.5, p = 0.676, r = 0.03). The composition of the top results differed by language: of the 44 English-language websites at positions 1 - 5, 14 (31.8%) were governmental websites (e.g., NHS, NIH/NINDS, MedlinePlus) and 20 (45.5%) were university or clinic websites, whereas only 3 of 178 German-language websites in the entire sample were governmental (Table 1). Rank-stratified results are shown in Table 5 and Fig. 4.

**Table 4:** Interrater reliability ICC (2,1).

Score	ICC (2,1)	Mean absolute difference	Median absolute difference	Spearman ρ
DISCERN	0.817	5.13	4	0.79
JAMA benchmarks	0.791	0.41	0	0.82

Intraclass correlation coefficient based on a two-way random-effects model with absolute agreement (Shrout & Fleiss). Values represent the agreement between Rater 1 and Rater 2 prior to score averaging.

**Table 5:** DISCERN and JAMA scores by Google search rank position and language.

Language/rank position	n	DISCERN, median (IQR)	DISCERN, mean ± SD	JAMA, median (IQR)
English, positions 1–5	44	49.8 (43.9–53.5)	48.4 ± 7.8	3.5 (2.5–4.0)
English, positions 6–10	45	42.0 (35.5–47.0)	40.7 ± 9.4	2.5 (2.0–3.5)
English, positions 11–15	43	40.0 (33.8–47.8)	39.8 ± 9.2	2.5 (2.0–3.2)
English, positions 16–20	45	41.0 (37.5–47.0)	41.5 ± 7.2	2.5 (2.0–3.5)
German, positions 1–5	45	44.0 (39.0–49.0)	43.3 ± 8.4	2.5 (2.0–3.5)
German, positions 6–10	44	35.5 (30.8–42.1)	36.1 ± 8.7	2.0 (1.5–3.0)
German, positions 11–15	45	42.0 (30.0–47.5)	38.7 ± 12.4	3.0 (2.0–3.5)
German, positions 16–20	44	41.8 (36.9–48.1)	40.7 ± 9.0	2.5 (1.5–3.5)

Rank position 1 denotes the first Google search result. English: Spearman ρ = −0.315 (DISCERN) and −0.178 (JAMA); German: ρ = −0.081 (DISCERN) and −0.066 (JAMA). English positions 1–10 vs 11–20: median DISCERN 45.5 vs 41.0, p = 0.002, r = 0.23; German: 40.5 vs 42.0, p = 0.676, r = 0.03.

**Fig. 4:**
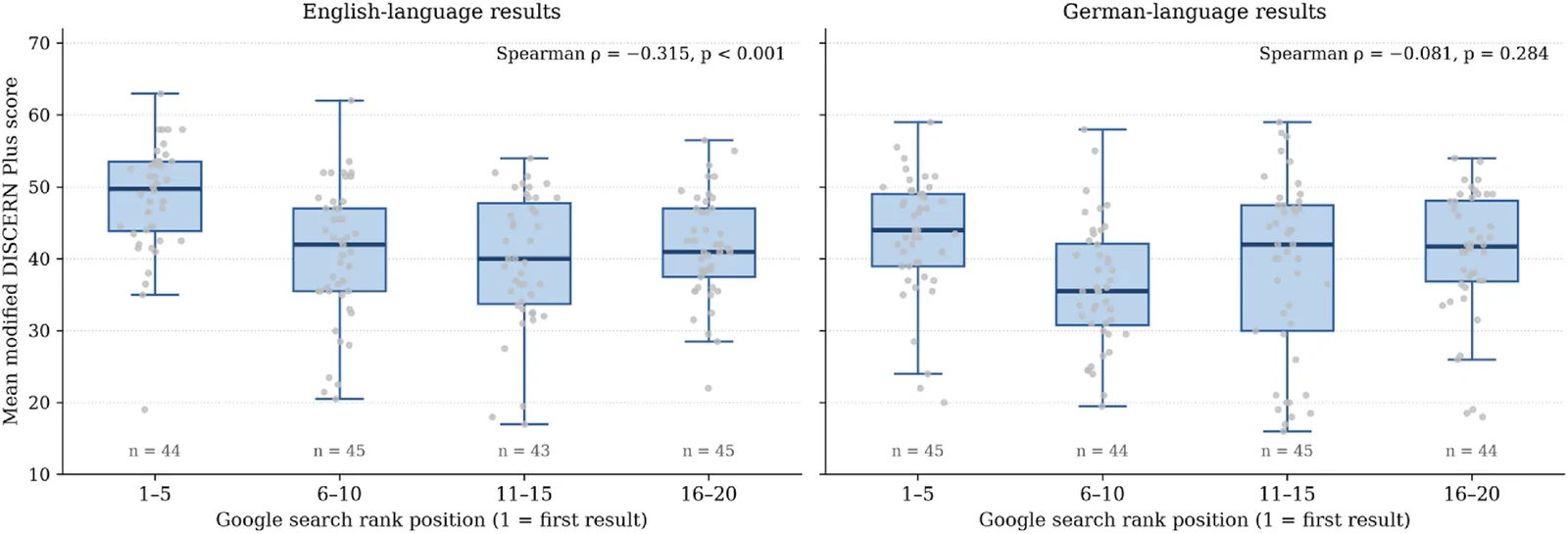
Mean modified DISCERN Plus scores by Google search rank position and language. Boxes show the median and interquartile range; whiskers show the 1.5 × IQR range; dots indicate individual websites; n per group is given below each box. Rank position 1 denotes the first search result. For English-language results, DISCERN scores decreased with increasing rank position (Spearman ρ = −0.315, p < 0.001), i.e., top-ranked websites were of higher quality, whereas no association was found for German-language results (ρ = −0.081, p = 0.284).

### 3.6 Correlation between DISCERN and JAMA scores

A moderate positive correlation was found between DISCERN and JAMA scores across all 355 websites (Spearman rho = 0.450, p < 0.001), indicating that websites scoring higher on the DISCERN reliability scale also tended to fulfill more of the JAMA transparency criteria.

### 3.7 Interrater reliability

Interrater reliability was good to excellent for both instruments. The ICC for DISCERN was 0.817 (two-way random-effects, absolute agreement), with a mean absolute difference between raters of 5.13 points and a Spearman correlation of 0.79. For JAMA, the ICC was 0.791 with a mean absolute difference of 0.41 points and a Spearman correlation of 0.82. These values are comparable to those reported in prior validation studies of both instruments (Rees et al., 2002; Cesur Aydin and Gunec, 2020). Full interrater data are provided in Table 4.

## 4 Discussion

This study provides a bilingual assessment of online patient information quality for eight vascular and skull base neurosurgical conditions searched under nine terms. Overall quality was in the “fair” range of the modified DISCERN Plus score, with variation across conditions, host categories and languages. The results extend the literature by covering several conditions without previous systematic quality analysis, including vestibular schwannoma, dural AV fistula, cerebral cavernoma, and Moyamoya disease.

The mean DISCERN score of 41.1 ± 9.7 lies between the “poor” average reliability reported for intracranial aneurysm websites (Patel et al., 2022) and the “good” average reported for brain AVM websites (Mehra et al., 2023). A meta-analysis of YouTube content on neurosurgical topics likewise found pooled DISCERN scores in the low-to-mid range (Váscones-Román et al., 2026). No website in our cohort was rated “excellent”, indicating a persistent quality ceiling in publicly available resources for these conditions.

English-language sites scored higher than German-language sites on both instruments, in line with a bilingual DISCERN Plus analysis of radiotherapy websites (Janssen et al., 2017) and with earlier observations on German-language health web content (Möller et al., 2023; Meyer et al., 2014; Zowalla et al., 2023). The effect sizes, however, were small (r = 0.12 - 0.13) and the median JAMA score was identical in both languages; the difference should not be over-interpreted, and its causes cannot be determined from our data.

For English-language results, top-ranked websites tended to be of higher quality (positions 1 - 5: median DISCERN 49.8 vs. 40.0 - 42.0 for positions 6 - 20). Since users predominantly click on the first results (Moccia et al., 2018), this is a favorable finding for English-speaking patients. It is largely explained by the composition of the top results: governmental resources (NHS, NIH/NINDS, MedlinePlus) and large academic centers dominated positions 1 - 5 of the English-language searches, whereas governmental websites were almost absent from the German-language results (3 of 178 websites). For German-language results, rank was not associated with quality, so that the position of a website offers German-speaking patients no guidance as to its reliability. Previous findings on search rank and quality are inconsistent: better quality at higher positions was reported for fracture-related websites (Memon et al., 2016), whereas Janssen et al. found an association only for German- and not for English-language websites (Janssen et al., 2017). Differences in search terms, languages and time points limit direct comparison.

Variation across search terms was small (ε^2^ ≈ 0.05), and no pairwise comparison remained significant after correction. Subarachnoid hemorrhage and intracranial aneurysm, conditions with established online patient communities (Boogaarts et al., 2014), scored highest, while the rarer entities dural AV fistula, cerebral cavernoma and Moyamoya disease scored lowest; whether this reflects a smaller volume of patient-oriented material for rare conditions cannot be determined from our data.

Host type explained about one quarter of the variance in JAMA transparency scores (ε^2^ = 0.248) but only a small, non-significant proportion of the variance in DISCERN scores (ε^2^ = 0.031). Educational and governmental websites scored highest on both instruments. University and clinic websites, the largest category and likely the most commonly visited by patients referred from clinical settings, scored at or below the overall median on both instruments; their low median JAMA score (2.0) indicates that at least two of the four transparency criteria were typically not fulfilled. As the individual JAMA criteria were not recorded separately, we cannot determine which were most frequently missing.

Several limitations should be acknowledged. First, the simulated search approach is not perfectly reproducible due to ongoing changes in search engine algorithms and web content; the searches reflect a single time window (March to May 2026). Second, the evaluation was limited to text-based web resources; video content, social media, and patient forums (Gajjar et al., 2024b; Szmuda et al., 2020) were not assessed. Third, DISCERN and JAMA assess the reliability and transparency of information rather than its readability or factual accuracy. We did not compare website content against clinical guideline recommendations; such an assessment was beyond the scope of this study, not least because recommendations differ between countries for several of the included conditions, but it remains an important objective for future work. Fourth, both raters had a medical background. Although DISCERN was designed to be applied by health consumers without specialist knowledge (Charnock et al., 1999) and interrater reliability was good, ratings by patients or by senior specialists might differ.

Despite these limitations, the study provides a large and methodologically consistent bilingual dataset covering two clinically important neurosurgical domains. Clinicians may consider providing patients with a curated list of high-quality online resources at the time of diagnosis, particularly German-speaking patients, for whom the position of a website in the search results is not indicative of its quality, and patients with rarer conditions.

## 5 Conclusion

Online patient information for vascular neurosurgery and skull base surgery conditions is predominantly of fair quality, with no site achieving an excellent rating. English-language websites scored higher than German-language websites, although the difference was small in magnitude, and website host type was strongly associated with transparency (JAMA) scores. In English-language searches, top-ranked results tended to be of higher quality, whereas search rank was not informative in German-language searches. These findings highlight a persistent gap between the information needs of patients facing complex neurosurgical diagnoses and the quality of resources that are most readily available to them. Efforts to improve the transparency of institutional websites and to guide patients, in particular German-speaking patients, towards evidence-based resources should be an integral part of the neurosurgical patient education strategy.

## Availability of data and material

The data supporting the findings of this study are available from the corresponding author on reasonable request.

## Code availability

Not applicable.

## Ethics approval

Not applicable. The study analyzed only publicly accessible online information and did not involve human subjects.

## CRediT author contribution statement

Janek Garber: Conceptualization, Methodology, Investigation (website evaluation and scoring), Formal analysis, Data curation, Writing – original draft, Visualization. Julia Bettina Kräutler: Methodology, Data curation, Writing – review & editing. Daniel Monden: Investigation, Writing – review & editing. Nadine Lilla: Resources, Writing – review & editing. Felix Behling: Validation, Writing – review & editing. Florian Gessler: Validation, Writing – review & editing. Ansgar Berlis: Resources, Writing – review & editing. Christian Senft: Resources, Supervision, Writing – review & editing. Dorothee Mielke: Supervision, Writing – review & editing. Paulina Günzerodt: Conceptualization, Methodology, Investigation (website evaluation and scoring), Formal analysis, Writing – original draft, Writing – review & editing, Project administration, Supervision. Peter Baumgarten: Conceptualization, Methodology, Supervision, Project administration, Writing – review & editing.

## Declaration of Generative AI and AI-assisted technologies in the writing process

During the preparation of this work the authors used Perplexity AI in order to improve the language and readability of the manuscript. After using this tool, the authors reviewed and edited the content as needed and take full responsibility for the content of the publication. All figures and tables are based on the authors’ original data.

## Funding

The research did not receive any specific grant from funding agencies in the public, commercial, or non-profit sectors.

## Declaration of competing interest

The authors declare the following financial interests/personal relationships which may be considered as potential competing interests: Florian Gessler reports a relationship with AstraZeneca Pharmaceuticals LP that includes: consulting or advisory, speaking and lecture fees, and travel reimbursement. Florian Gessler reports a relationship with QV Bioelectronics that includes: consulting or advisory. Florian Gessler reports a relationship with B. Braun Medical Inc. that includes: consulting or advisory, speaking and lecture fees, and travel reimbursement. Florian Gessler reports a relationship with signus spine that includes: speaking and lecture fees and travel reimbursement. Florian Gessler reports a relationship with Richard and Annemarie Wolf Foundation that includes: funding grants. If there are other authors, they declare that they have no known competing financial interests or personal relationships that could have appeared to influence the work reported in this paper.
