# Supplementary material for: Assessing the quality of online resources for vascular neurosurgery and skull base surgery: A bilingual evaluation using DISCERN and JAMA benchmarks
Source: Brain Spine. 2026 Nov 2;6:107718. doi: 10.1016/j.bas.2026.107718 (PMC13756516; doi:10.1016/j.bas.2026.107718)
Supplement: Supplementary file 1 [file mmc1.docx]

**Standardized Chart for the Quality Assessment of Websites**

Domain: ___________________________________

Google hit number: _______________

**Category**

| □ | Medical news site (sponsored) |
| --- | --- |
| □ | Medical center/university |
| □ | Interest group/NGO |
| □ | Governmental |
| □ | Educational |
| □ | Other |

**JAMA Benchmarks (yes/no) (Silberg et al. 1997)**

| □ | Authorship: authors/contributors/affiliations |
| --- | --- |
| □ | Attribution: References/sources/copyright |
| □ | Disclosure: ownership/sponsors/support |
| □ | Currency: creation/update date |

**Total JAMA score:** ______ / 4

**Modified DISCERN Score (Charnock et al. 1999)**

**Section 1 – General quality of health information (1–5 points each)**

| **#** | **Item** | **Guidance** | **Pts** |
| --- | --- | --- | --- |
| 1 | **Are the aims clear?** | Are the objectives of the website clearly defined? What is the subject matter? Which topics are addressed? Who is the target audience? |  |
| 2 | **Does it achieve its aims?** | Does the website achieve its self-imposed goals? |  |
| 3 | **Is it relevant (reader's perspective)?** | Are questions addressed that readers would ask, using understandable language? |  |
| 4 | **Sources of information** | Are the information sources used clearly indicated? How many references are listed? Is the author clearly identified? |  |
| 5 | **Up-to-datedness of data** | Is the creation date, update date, and dates of references clearly indicated? |  |
| 6 | **Balanced and unbiased?** | Is the presentation balanced and objective? Are multiple sources cited? Is external review mentioned? |  |
| 7 | **Additional sources?** | Does the website contain information about supplementary aids and resources? |  |
| 8 | **Refer to areas of uncertainty?** | Are different possibilities addressed? Is lack of evidence mentioned? Is it indicated that information should not be used for self-diagnosis? |  |

Section 1 subtotal: ______ / 40

**Section 2 – Condition-specific questions (1–5 points each)**

| **#** | **Item** | **Guidance** | **Pts** |
| --- | --- | --- | --- |
| 9 | **Consequences/risks of [SEARCH TERM]** | Does the website describe the consequences or risks associated with [SEARCH TERM]? |  |
| 10 | **When to contact a physician** | Does the website provide recommendations on when to contact a physician regarding [SEARCH TERM]? |  |
| 11 | **Treatment options** | Does the website describe possible treatment options for [SEARCH TERM] and the mechanism of action of each? |  |
| 12 | **Risks of treatment** | Does the website describe the risks associated with each treatment procedure for [SEARCH TERM]? |  |
| 13 | **Diagnostic procedure** | Does the website describe the diagnostic procedure for [SEARCH TERM]? |  |
| 14 | **Quality of life / treatment setting** | Does the website describe how treatment for [SEARCH TERM] affects quality of life or the setting in which therapy is provided? |  |
| 15 | **Pre- or post-treatment recommendations** | Are recommendations made regarding pre- or post-treatment procedures for [SEARCH TERM]? |  |

Section 2 subtotal: ______ / 35

**Section 3 – Overall rating of the publication**

| **#** | **Item** | **Guidance** | **Pts** |
| --- | --- | --- | --- |
| 16 | **Overall quality** | Based on all preceding questions, evaluate the overall quality of this website as a source of information about [SEARCH TERM]. Rate on a scale of 1 (very low quality) to 5 (very high quality). |  |

**Total Modified DISCERN Score:** ______ / 80

**Exclusion Criteria**

| □ | Limited information on search topic (< one paragraph) |
| --- | --- |
| □ | Complete access restricted by password |
| □ | Repeated server unavailability |
| □ | Direct forwarding to other domains |
